# Wearable Biosensor with Molecularly Imprinted Conductive Polymer Structure to Detect Lentivirus in Aerosol

**DOI:** 10.3390/bios13090861

**Published:** 2023-08-31

**Authors:** Jaskirat Singh Batra, Ting-Yen Chi, Mo-Fan Huang, Dandan Zhu, Zheyuan Chen, Dung-Fang Lee, Jun Kameoka

**Affiliations:** 1 Department of Materials Science and Engineering, Texas A&M University, College Station, TX 77840, USA; jbatra@tamu.edu (J.S.B.); kevin0149@tamu.edu (T.-Y.C.); 2 Department of Integrative Biology and Pharmacology, McGovern Medical School, The University of Texas Health Science Center at Houston, Houston, TX 77030, USA; mo-fan.huang@uth.tmc.edu (M.-F.H.); dandan.zhu@uth.tmc.edu (D.Z.); dung-fang.lee@uth.tmc.edu (D.-F.L.); 3 The University of Texas MD Anderson Cancer Center UTHealth Graduate School of Biomedical Sciences, Houston TX 77030, USA; 4 Department of Electrical and Computer Engineering, Texas A&M University, College Station, TX 77843, USA; zychen@tamu.edu; 5 Graduate School of Information, Production and System Research, Waseda University, Fukuoka 808-0135, Japan

**Keywords:** wearable paper sensor, molecular imprinting, conductive polymer, lentivirus, virus sensor

## Abstract

The coronavirus disease (COVID-19) pandemic has increased pressure to develop low-cost, compact, user-friendly, and ubiquitous virus sensors for monitoring infection outbreaks in communities and preventing economic damage resulting from city lockdowns. As proof of concept, we developed a wearable paper-based virus sensor based on a molecular imprinting technique, using a conductive polyaniline (PANI) polymer to detect the lentivirus as a test sample. This sensor detected the lentivirus with a 4181 TU/mL detection limit in liquid and 0.33% to 2.90% detection efficiency in aerosols at distances ranging from 30 cm to 60 cm. For fabrication, a mixture of a PANI monomer solution and virus were polymerized together to form a conductive PANI sensing element on a polyethylene terephthalate (PET) paper substrate. The sensing element exhibited formation of virus recognition sites after the removal of the virus via ultrasound sonication. A dry measurement technique was established that showed aerosol virus detection by the molecularly imprinted sensors within 1.5 h of virus spraying. This was based on the mechanism via which dispensing virus droplets on the PANI sensing element induced hybridization of the virus and molecularly imprinted virus recognition templates in PANI, influencing the conductivity of the PANI film upon drying. Interestingly, the paper-based virus sensor was easily integrated with a wearable face mask for the detection of viruses in aerosols. Since the paper sensor with molecular imprinting of virus recognition sites showed excellent stability in dry conditions for long periods of time, unlike biological reagents, this wearable biosensor will offer an alternative approach to monitoring virus infections in communities.

## 1. Introduction

The global health threat from COVID-19 has raised attention regarding the need for low-cost, rapid, sensitive, compact, and selective detection platforms for viruses. In particular, keeping viral diseases from spreading into the community relies heavily on the detection of such viruses at the initial stages of an outbreak. Individual personal virus testing and aerosol virus detection can trace the spread of infection, minimizing virus outbreaks and preventing city lockdowns. Currently, virus detection relies on conventional approaches such as serological [1] and viral nucleic acid tests [2]. Serological tests include antibody and antigen detection in immune chromatography formats. Antibody detection involves detecting antibodies produced by the immune system once it is exposed to a virus. This approach often shows false-negative results for actively infected persons. Antigen testing uses the same platform as antibody testing and can rapidly detect virus markers that are virus surface proteins. This serological platform is mostly built on a paper substrate with a sample pad, gold-conjugated pad, capture/test line, and control line [3]. Once the sample solution is dispensed onto the sample pad, target molecules (either antigens or antibodies) are conjugated with target antibodies bound with gold nanoparticles. Molecular complexes are also conjugated with antigen/antibody-specific antibodies at the testing line, visually indicating the existence of antibodies/antigens. These approaches are generally less sensitive than nucleic acid tests and can show false-negative results at the early stages of diagnosis. Currently, quantitative reverse transcription polymerase chain reaction (qRT-PCR), a viral nucleic test, is the gold standard for detecting viruses. It operates by amplifying virus DNA or RNA after extracting a virus gene, and then measuring the fluorescence intensities from the amplified virus gene to detect the virus. Many commercial qRT-PCR kits are available, even for COVID-19 [4]. The drawback of this approach is that it is time-consuming, demands high costs for reagents and bulky detection equipment, and requires trained lab personnel [5].

In an effort to reduce sensing cost, improve convenience, and provide a more compact design, various new virus sensing approaches have been put forth. Electrochemical detection through low-cost paper substrates is one of the most popular for detecting viruses [6,7]. Reference, counter, and working electrodes are screen-printed on paper or polymer substrates, and reagents such as antibodies or aptamers immobilized on the working electrode. Hybridization of target molecules on working electrodes via a reagent induces an impedance change between the reference and working electrodes. The detection of viruses such as H1N1 [8,9,10], H5N1 [11], SARS-CoV-2 [12], and H7N9 [11,13,14] has been demonstrated using this approach. However, electrochemical detection still requires costly reagents such as antibodies, peptides, and aptamers, as well as makes it challenging to detect aerosol viruses.

To replace the costly biological reagents, molecularly imprinted electrodes for electrochemical detection have also been suggested. Instead of the metal or carbon working electrode, a conductive polymer electrode with target molecule templates was used to detect the Zika virus [15,16]. In this process, the Zika virus was molded onto the surface of a graphene oxide (GO) polymer composite solution and templates formed by eliminating the virus from the composite for detection purposes. Hybridization of the virus on the working electrode influenced the electrochemical impedance. The molecular imprinting approach has also been shown to detect other viruses such as water-borne viruses [17], influenza A [18,19], HIV [6], Japanese encephalitis [20], dengue virus [21], and hepatitis C [22]. The drawback of the molecularly imprinted polymer (MIP) electrochemical approach is that it still requires bulky potentiostat detection equipment, and detection requires a liquid environment. Thus, it is still challenging to detect viruses under dry conditions and in aerosol formats.

The present research demonstrated a low-cost MIP lentivirus sensor that requires no biological reagent and enables virus detection in liquid and aerosol phases without bulky equipment. We previously developed glucose and perfluorooctanesulfonic acid (PFOS) conductive molecularly imprinted paper sensors [23,24,25] and expanded this approach to the detection of viruses. Lentivirus was used as a proof-of-concept test sample because it can be used in biosafety level 2 labs and is considered a safe alternative to live coronavirus. To form the molecular imprinting template for lentivirus, the virus was blended with a conductive monomer solution polymerized on a polyethylene terephthalate (PET) paper substrate. The lentivirus was then removed from the molecularly imprinted template by ultrasonication. Edges of the conductive MIP sensing paper were connected using two copper metal tapes as electrodes to measure the conductance of the MIP sensing structures. Since the electrical resistance of the conductive polymer polyaniline (PANI) relied on the polaron hopping through π–π stacking, the absorption of virus in the MIP sensing structure modulated the conductance of the paper sensor.

We established a dry measurement technique for virus detection that includes the process of the virus and molecularly imprinted cavity hybridization and sensor element drying. The electrical resistance of the sensor, fabricated from the conductive polymer, enabled the passive measurement of the resistivity ratio over time through the use of a portable multimeter. With this approach, we were able to demonstrate detection of the lentivirus on the sensing element (i) within 1 h by simply dispensing liquid, and (ii) within 1.5 h after spraying a virus solution. This simple and low-cost approach can potentially detect viruses from liquid and aerosol samples in a short period of time. To the best of our knowledge, this is the first attempt to demonstrate the detection of viruses directly in aerosols. The stability of the molecularly imprinted structure in the air [24] will possibly reduce outbreaks and prevent large-scale lockdowns in the future.

## 2. Materials and Methods

### 2.1. Materials

Aniline and ammonium persulfate (APS) acting as the monomer and the oxidant, along with phosphate-buffer saline (PBS) for adjusting the virus concentration, were purchased from Sigma-Aldrich (St. Louis, MO, USA). Acidic stock solutions including hydrochloric acid (HCl, 36–38%) and acetic acid were acquired from Macron (Center Valley, PA, USA). Methanol was ordered from VWR Chemicals (Radnor, PA, USA). Polyethylene terephthalate paper and silver conductive paste (Cat# 125-15) were provided by Xerox (Norwalk, CT, USA) and Creative Materials (Ayer, MA, USA), respectively.

The generation of lentiviruses was described previously [26], and the detailed protocol is included in the Supplemental Materials of this paper. Briefly, HEK-293T cells were maintained in DMEM supplemented with 10% (vol/vol) Opti-Gold fetal bovine serum (FBS, GenDEPOT, Katy, TX, USA), L-glutamine, and penicillin/streptomycin. pLKO.pig, pCMV-VSVG (envelope, Addgene plasmid #8454), and pCMV-dR8.2 (packaging; Addgene plasmid #8455) plasmids were co-transfected into HEK-293T cells by the PEI transfection reagent. After 18 h transfection, the fresh medium was replaced. At day 3 post transfection, the supernatant containing lentiviral particles was collected. The entire synthesis was performed under a biosafety level 2 environment. The virus concentration of the stock vial was estimated to be 2.2 × 10^5^ TU/mL, where TU denotes the transducing units of viral particles. Retro-CMV-GFP retroviruses (RVP003, Applied Biological Materials, Vancouver, BC, Canada), polystyrene latex beads (0.1 μm mean particle size, Sigma-Aldrich, St. Louis, MO, USA), and human whole blood (stocked in EDTA K2, BioChemed Services, Winchester, VA, USA) were used as selectivity references. Phosphate-buffered saline (PBS) for adjusting the virus concentration was diluted from concentrated PBS (10×) purchased from Sigma-Aldrich (St. Louis, MO, USA).

### 2.2. Synthesis of Virus-Imprinted Polyaniline Structure

The synthesis of the virus-imprinted PANI followed the protocol detailed in the previous study [24]. Briefly, 200 μL of aniline and 500 μL of the virus stock solution (2.2 × 10^5^ TU/mL) as the molecular imprinting template were blended into 1 M HCl with a final volume of 5 mL as the monomer solution. The final virus concentration in the polymer solution was 1.1 × 10^4^ TU/mL. Paper strips (1 cm × 0.5 cm) were dipped into the monomer solution for 5 min to saturate the solution on the paper. The oxidant solution prepared by mixing 409 mg of APS with 5 mL of 1 M HCl was then added drop by drop to initiate polymerization for 10 min. After the bulk polymerization, the PANI strips were taken out from the solution and rinsed with deionized water until the eluent showed no excess PANI particles. The virus templates were removed by sonicating the strips in acetic acid/methanol solution (vol/vol = 1:6) for 4 h, followed by rinsing the strips until the pH value of the eluent reached to 7. The resulted lentivirus molecularly imprinted (MIP) PANI paper strips were air-dried at ambient temperature overnight. The monomer solution without lentiviruses was synthesized as non-molecularly imprinted (NIP) control devices. NIP preparation without virus followed the same treatment as MIP.

### 2.3. Fabrication of Virus MIP Paper Sensor

To fabricate the duplex sensor device, one NIP and another MIP strip were integrated with a plastic stencil substrate (2 cm × 2.5 cm) using double-sided tape, with copper tape (1 cm × 0.635 cm) as the contact electrode. The strip and electrode were separated by a 1 mm gap which was then filled with silver conductive paste. The silver paste was cured at 25 °C for at least 12 h to ensure optimal conductivity, and the resulting device was used to measure the resistivity responses of NIP and MIP to lentiviruses on a single device. The surfaces of NIP and MIP PANI were investigated by scanning electron microscopy, and the result is shown in Supplement 5.

### 2.4. Lentivirus Detection and Resistance Measurement

To test the detection efficacy of virus MIP sensor, virus samples with different concentrations were prepared. Specifically, for the selectivity test, the virus concentration of lentivirus and Retro-CMV-GFP retrovirus was set at 1.65 × 10^5^ TU/mL. Then, an aliquot of 30 μL virus solution was dispensed in the middle of the PANI sensing element (0.5 cm × 0.5 cm) and saturated for 30 min, followed by gentle aspiration and air-drying at 25 °C for 30 min. For virus detection in aerosol, virus contained in DMEM solution was sprayed onto the PANI sensing element, as described in detail in Supplement 6, and the resistance measurements were conducted.

To obtain the virus concentration calibration curve, the resistance of the PANI electrode was measured using a multimeter (8846A, Fluke, Everett, WA, USA) in direct current mode after a drying time of 30 min. This was the time needed to air-dry the sensor surface after 30 µL of virus solution was dispensed and aspirated until no visible moisture was found. Resistance was converted to resistivity as shown in Equation (1).

(1) $$\rho =\frac{R\;A}{L}$$

In this equation, *A* and *L* are the cross-sectional area and the longitudinal length of the PANI sensing element, respectively. In addition, the resistivity after virus exposure was divided by the resistivity before virus exposure to determine the resistivity ratio which is the output signal of the sensor calculated using Equation (2) [24].

(2) $$Resistivity\;Ratio\;(RR)=\frac{{\rho \;}_{after\;virus}}{{\rho \;}_{before\;virus}}$$

To investigate the effect of non-specific bonding, the resistivity ratio of NIP were subtracted from that of MIP as shown in Equation (3).

Specific binding = RR_MIP_ − RR_NIP_ (3)

The limit of detection (*LoD*) was estimated using Equation (4), where the slope (*m*) was obtained from the linear regression fit of specific binding. *σ* is the standard error of blank samples that were exposed to PBS [27].

(4) $$LoD=\frac{3\sigma }{m}$$

To study the stability of the PANI electrode, PANI was first synthesized on paper specimens (1 cm × 1 cm) using the same synthetic protocol above. The resistance was then measured weekly at room temperature (25 °C) for 13 weeks after the fabrication (total of 14 weeks), and the normalized resistivity was calculated.

## 3. Results and Discussion

### 3.1. Calibration Curve: Lentivirus Concentration from Liquid on Virus MIP Sensors

**Figure 1:**
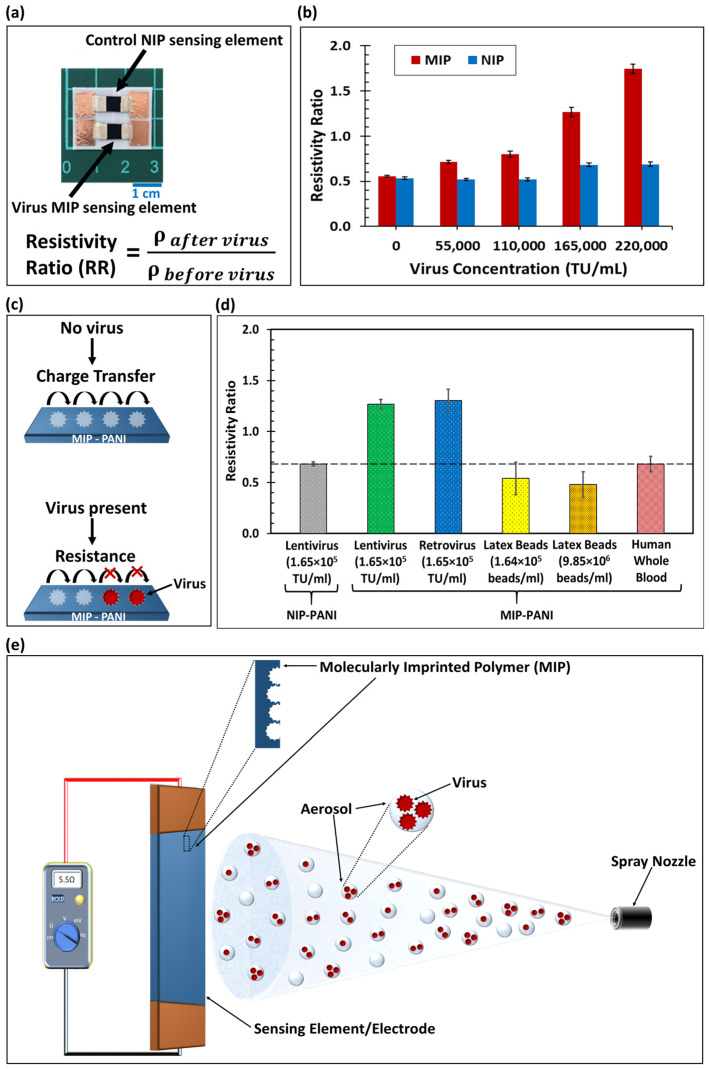
(**a**) Photographic image of virus sensor with MIP and NIP electrodes. Scale is 1 cm. (**b**) Resistivity ratio of the MIP and NIP electrodes as a function of the lentivirus concentration (*n* = 3, RSD avg. = 3.0%). For calibration, the lentivirus was detected after a 30 min liquid saturation, aspiration, and 30 min natural drying process. (**c**) Schematic of virus detection mechanism showing polaron hopping and charge transfer in MIP-conductive PANI. The electrical resistance increased from the virus present in the molecularly imprinted cavity. (**d**) Selectivity test of MIP electrode and comparison to NIP control (represented by dashed line). The resistivity ratios of the lentivirus, retrovirus, latex beads, and human whole blood were calculated from resistance measurements (*n* ≥ 5, RSD avg. = 13.7%). (**e**) Diagram of MIP sensing electrode with molecularly imprinted cavities inside the conductive polymer and virus aerosol sprayed onto the sensing element. TU = transducing units; RSD = relative standard deviation.

The resistivity ratios of the MIP and non-molecularly imprinted polymer (NIP) electrodes (as shown in Figure 1a) after being exposed to the lentivirus in liquid form and dried for 30 min are plotted as a function of virus concentration in Figure 1b. After sensor drying, the 24 h observation of resistance showed stable values, as displayed in Supplement 7. The I–V curve for this sensor is shown in Supplement 8.

The MIP and NIP resistivity ratios were identical at zero virus concentration. As the virus concentration increased, the MIP electrode showed an increase in the resistivity ratio (Figure 1b), which was much larger than the NIP value. PANI conductivity relies on polaron hopping through π–π stacking, as shown in Figure 1c, which is generated by acidic doping in its emeraldine form. When the negatively charged viruses in a neutral pH condition [28] were captured on the MIP electrodes, the virus particles electrostatically neutralized the polaron hopping in the electrode (see Figure 1c). Therefore, this virus-capturing process obscured the charge transfer route, increasing the resistivity of the MIP electrode.

In the experiment, the NIP resistivity ratio remained constant for lentivirus concentrations up to 1.1 × 10^5^ TU/mL (TU = transducing units of viral particles). At high lentivirus concentrations (1.65 × 10^5^ TU/mL and 2.2 × 10^5^ TU/mL), there was a slight increment in the NIP resistivity ratio. However, this extremely small signal increment (attributable to non-specific binding) was removed from the overall sensor signal (see Supplement 1, Figure S1 in Supplementary Materials). The limit of detection for lentivirus sensing by the MIP sensor was estimated to be 4181 TU/mL (or 125 TU virus particles). The sensor made from polyaniline was stable at temperatures between 11.0 °C and 41.5 °C and relative humidities ranging from 15% to 85% for 11 weeks (see Supplement 2, Figures S2–S4).

Even though the MIP sensor demonstrated a promising virus detection efficacy as compared to the NIP control, a small increase in the non-specific binding of the lentivirus was found on the NIP electrode at high virus concentrations. This could be due to the lentivirus–polyaniline material interactions and some of the virus adhering to the surface, despite the absence of molecularly imprinted templates in the NIP electrode (since the PANI surface was not smooth). To eliminate such bias from the total sensor response, the subtracted (i.e., MIP–NIP) data were essential, accounting for the specific recognition of the lentivirus by the MIP sensor. The significant difference in resistivity ratio between the MIP and NIP responses supports the strategy of molecular imprinting technology.

The lentivirus served as an effective template for fabricating the molecular imprinting virus sensor. The sensor’s virus-capturing efficiency and detection limit could be further improved by increasing the concentration of the virus used for fabrication, which would amplify the number of MIP cavities. Further surface investigation is needed to visualize the functional groups and intermolecular interactions between the virus particles and MIP polyaniline matrix. Studies of the viability and possible structural transition of the lentivirus during synthesis are also necessary to reveal the detailed mechanisms of molecular imprinting associated with microorganisms.

### 3.2. Sensor Selectivity

We investigated the selectivity of the MIP sensor by comparing the resistivity ratios of the lentivirus, retrovirus, latex beads, and human blood. The results are shown in Figure 1d. The MIP sensor demonstrated a promising selectivity for detection of the lentivirus, as compared to other nanoparticles and whole blood.

The MIP sensor detected the Retro-CMV-GFP retrovirus (1.65 × 10^5^ TU/mL virus concentration), in addition to the lentivirus, since the resistivity ratios on the MIP sensors were similar. Because the lentivirus and retrovirus belong to the same virus family and their viral envelope structures are almost identical, the retrovirus was also captured by the MIP structure synthesized with the lentivirus template. This shows the ability of the sensor to capture the same family of viruses. Future investigations will compare the selectivity of different families of viruses.

Moreover, the detection of 100 nm polystyrene latex beads roughly the same size as the lentivirus were investigated at the same concentration of 1.64 × 10^5^ beads/mL as an analogue of virus particles. The resistivity ratio for the MIP electrodes with regard to latex bead detection showed no significant difference from the NIP control results. Even with a much higher concentration of beads (9.85 × 10^6^ beads/mL, 60 times the virus concentration), no difference from the control in terms of resistivity ratio was observed. This confirmed that the MIP electrode did not respond to the beads, even though they were the same size as the virus. Additionally, no significant MIP resistivity change was found with the human whole blood sample containing potential contaminants such as plasma and blood cells. The resistivity ratio for the blood sample on the MIP electrode was close to that of the NIP control. Even though the geometric shapes of polystyrene nanoparticles and blood proteins are roughly spherical, similar to the lentivirus shape, they may lack the surface interactions found in the lentivirus. The absence of surface interactions with the molecularly imprinted cavities may have prevented nanoparticles and blood proteins from being captured on the molecularly imprinted sensor.

Due to the presence of MIP cavities for virus capturing, this sensing device exhibited more substantial responses to the lentiviruses than did the NIP device. Although the strongly acidic condition of the monomer solution remains a concern with regard to preventing the lentiviruses from maintaining viability, the geometric structure may still be maintained during the molecular imprinting process, since it is of a short duration. More characterizations are needed to acquire further evidence and visualize the formation related to the molecularly imprinted structures.

### 3.3. Lentivirus Detection from Aerosol on Virus MIP Sensors

**Figure 2:**
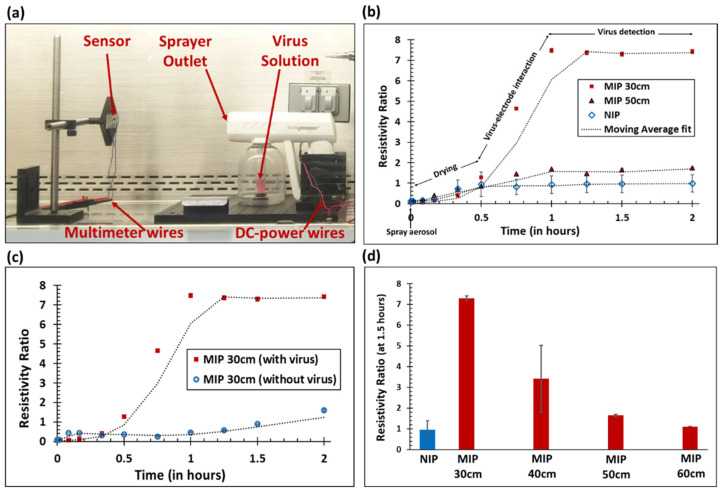
Resistivity ratio as a function of time and distance (*n* ≥ 5, RSD avg. = 13.3%). Lentivirus aerosol was sprayed for 10 to 12 s (initial volume of lentivirus solution = 2.65 ± 0.15 mL; approximate virus concentration = 2.2 × 10^5^ TU/mL). (**a**) Photographic image of the aerosol sprayer and virus detection using sensing electrodes. (**b**) Resistivity ratios for the MIP and NIP sensing elements as a function of time. (**c**) Resistivity ratio for the MIP sensing element with and without the virus. DMEM was used as a control without the virus. (**d**) The resistivity ratio (time = 1.5 h) for the MIP sensing element as a function of sprayer distance. The resistivity ratio for the NIP control was averaged across various distances.

The resistivity ratio of the MIP sensor for the lentivirus in aerosol form was measured from multiple spray distances (between 30 cm and 60 cm) to investigate the feasibility of the sensor (detailed procedure shown in Supplement 6). The conceptual diagram and experimental setup are shown in Figure 1e and Figure 2a, respectively. The results of the resistivity ratio as a function of time with different distances are shown in Figure 2b.

The resistivity ratio increased over time and reached the saturation value at which the resistivity ratio detects viruses. In this graph, from 0 to 0.5 h, the resistivity ratio increased slightly, which can mainly be attributed to the drying of the sensing surface that was wetted by the aerosol particles. A sharp increase in the sensor response from 0.5 to 1 h was observed. This was primarily because of hybridization of the virus with the MIP templates; there was very little contribution from the drying of the sensor. According to the humidity experiment (see Figure S4), the sensor resistivity remained constant for a relative humidity between 15% and 85%. In Figure 2b, the resistivity ratio increased over time, as the conductive polymer’s electrical path was obstructed by the virus. After 1 h, the resistivity ratio reached saturation because all of the virus particles present on the surface were electrically detected by the molecularly imprinted conductive polymer and the surface was visibly dried. The NIP control electrode showed very little response to the lentivirus aerosol (as compared to the MIP sensing electrode) at 30 cm and 50 cm, which is consistent with lentivirus detection in liquid. Further, the resistivity ratio of the MIP sensor at 30 cm was significantly higher than at 50 cm. This was due to the higher volume of aerosol virus deposited on the sensing element since the distance between the sensor and sprayer outlet was shorter. The NIP resistivity ratio value over various distances was averaged, resulting in a higher standard deviation of NIP (see Figure 2b). When the MIP sensing element sprayed with the lentivirus was compared to the MIP sprayed with DMEM without the virus, the resistivity ratio was significantly higher in the presence of virus particles after saturation (see Figure 2c). This further exemplifies the functionality of the MIP sensor in the presence of the virus.

Since the resistivity ratio remained constant after 1 h, a fixed time of 1.5 h was used to compare the resistivity ratio as a function of distance (see Figure 2d). The MIP resistivity ratios at a distance between 30 cm and 60 cm decreased as the sensor moved further away from the sprayer. This was due to the reduction in virus aerosol volume reaching the sensing element as the distance was increased. At a short distance from the sprayer (less than 30 cm), a very high volume of virus aerosol was sprayed, resulting in excessive wetting of the sensing electrode, not a realistic situation. Therefore, a minimum distance of 30 cm was selected. Far away from the sprayer (up to 60 cm), a very small amount of virus aerosol reached the MIP sensing electrode, resulting in a resistivity ratio that approached the NIP control value. On the basis of this result, we concluded that the lentivirus could be detected in an aerosol format using the virus MIP sensor at distances between 30 cm and 60 cm from the virus source.

In Figure 2d, the MIP resistivity ratio at 40 cm had a large standard deviation, which could be due to variations in the total volume of aerosol particles reaching the electrodes at this distance. Even though the maximum distance of the aerosol spray was 60 cm, the aerosol distribution in the air from 30 cm to 60 cm was not uniform, due to fluidic instability [29]. At a critical distance of 40 cm away from the sprayer, the fluctuation in aerosol volume reaching the electrodes may have affected the resistivity ratio measurements, leading to a large standard deviation. To verify, the aerosol spray characteristics were visualized using a colored dye sprayed on white paper (see Supplement 3, Figure S5). Additionally, compared to lentivirus detection in liquid at 1 h (30 min of liquid saturation, gentle aspiration, and an additional 30 min of drying time), lentivirus detection in aerosol form required a slightly longer duration of 1.5 h, due to the additional time needed to dry the sensing elements. Detection of virus aerosol using the passive resistance method discussed in this paper simplifies the virus detection process significantly as compared to the costly electrochemical method; however, the trade-off is a longer detection time due to complete drying required for virus effects to become evident in the MIP sensing layer. This detection time using resistance measurement could be significantly reduced using a faster convective evaporation approach. The evaporation rate for the MIP sensor and its impact on the resistivity ratio will need to be further studied in detail in the future.

To confirm lentivirus detection in aerosol form, the resistivity ratio of the lentivirus sprayed at 50 cm was compared with the calibration curve trendline fit (see Figure S1) and the amount of lentivirus calculated. Using the MIP and NIP resistivity ratios, the number of lentivirus particles detected in aerosol at 50 cm was found to be 5275 TU. Since the total number of particles at the spray source was 5.8 × 10^5^ TU, this meant that only 0.90% of the lentivirus was detected by the sensor at a 50 cm distance. As a function of the spraying area characteristics (see Figure S5), the theoretical amount of lentivirus reaching the sensing element was calculated to be 74,566 TU, assuming all aerosols from the sprayer reached the 50 cm sensing position. Even though 12.79% of the total virus reached the sensing element area, only 0.90% of the total virus was electrically detected. This calculation resulted in a 7.07% hybridization efficiency from the 50 cm spray distance. This could have been due to the specific detection of only those viruses on the sensor trapped inside the molecularly imprinted cavities. From the 30 cm to 60 cm sprayer distance, the virus detection efficiency was found to be between 0.33% and 2.90% (see Figure S6). At 60 cm, the amount of lentivirus calculated from calibration curves was 1953 TU, which approached the limit of detection (4181 TU/mL) for this sensor.

### 3.4. Face Mask Application and Sensor Accuracy, Sensitivity, and Specificity

**Figure 3:**
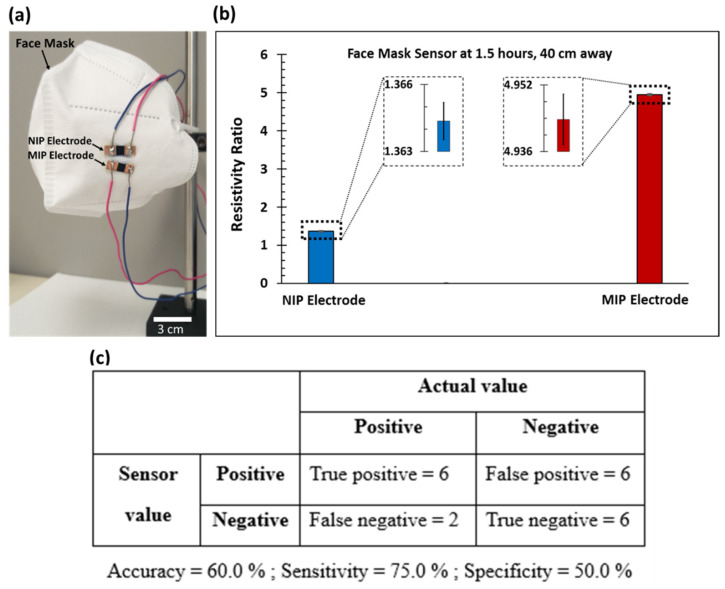
Lentivirus detection using face mask virus sensor. (**a**) Image of the face mask sensor with NIP and MIP electrodes attached using epoxy. The scale bar is 3 cm. A video is available for download from the Supplemental Materials. (**b**) The resistivity ratio (time = 1.5 h) of the NIP and MIP electrodes when the face mask was placed 40 cm away from the aerosol sprayer (*n* = 5, RSD avg. = 0.1%). The inset shows the error bars. (**c**) Diagnostic test parameters for the virus MIP sensor (*n* = 20).

A wearable virus sensor application was demonstrated by attaching the NIP and MIP sensing electrodes to a face mask to detect the lentivirus in aerosol form (see Figure 3a). The maximum dimensions of the face mask were 16 cm × 11 cm.

At a detection time of 1.5 h and with the face mask sensor placed 40 cm away from the aerosol sprayer, the resistivity ratios of the NIP and MIP electrodes were found to be 1.364 ± 0.001 and 4.944 ± 0.006, respectively (as shown in Figure 3b), with a virus concentration of 2.2 × 10^5^ TU/mL. The resistivity ratio of this sensor was within the standard deviation at a 40 cm distance (see Figure 2d). This 40 cm distance for the face mask experiment was selected because it is a similarly close distance to two people standing next to each other. The fluctuation in aerosol volume reaching the face mask sensor electrodes at 40 cm, which is the critical spray distance, could have caused the MIP_face mask_ value to be at the extreme end of the MIP_40 cm_ aerosol detection curve.

Using the specific binding curve (see Figure S1 and Equation (S8)), the amount of lentivirus on the face mask sensor was calculated to be 12,600 TU. This meant that 2.16% of lentivirus was detected by the face mask sensor at 40 cm, as compared to the total amount of virus. With 13.74% of the total virus aerosol reaching a sensor element placed at a 40 cm distance, a virus hybridization efficiency of ~15.73% for the face mask sensor was calculated. The virus detection efficiency at 40 cm was approximately twice the value for a sensor placed at a 50 cm distance (i.e., 2.16% vs. 0.90%). Overall, the virus detection efficiency decreased as the sensor was placed further away from the sprayer, which is illustrated in Figure S6.

A preliminary determination of diagnostic test parameters for the virus MIP sensor was achieved by comparing the MIP sensor output with the known experimental conditions (presence or absence of the virus, control, etc.). Figure 3c is a summary of the sensor parameters resulting in 60.0% accuracy, 75.0% sensitivity, and 50.0% specificity, illustrating the potential of the low-cost virus MIP sensor to provide clinically useful results with relatively high accuracy and sensitivity. Additional investigations with a larger sample size are needed to confirm the diagnosis of a virus using this aerosol sensor.

While the importance of aerosol virus detection is enhanced by the urgent need to slow down and prevent the respiratory transmission of infectious viruses such as COVID-19 [30], there is still a lack of tools available to efficiently capture viruses from aerosols [31]. Our virus MIP sensor integrates the virus capturing and detection processes in a single platform. To improve the virus detection efficiency from aerosol particles on the sensor surface, a highly porous membrane or molecular absorbent layer made from a metal–organic framework [32] could be used on top of the molecularly imprinted polymer sensor. Viruses in electrostatically charged aerosols could further be attracted to the sensing surface by applying a small voltage near the sensor region, which would further enhance the virus capture efficiency of the sensor.

## 4. Conclusions and Future Directions

We designed a low-cost paper-based virus sensor with a detection limit of 4181 TU/mL using the molecularly imprinted polymer technique. We also demonstrated virus detection in aerosols using our paper-based MIP sensor, as well as a wearable sensor application in the form of a face mask, which will be critical in future public health and safety. In the aerosol experiments, we detected the lentivirus at distances ranging from 30 cm to 60 cm away from the virus sprayer. The sensor showed a virus detection efficiency of 0.33% to 2.90% from the lentivirus present in the aerosol. The dry measurement of the virus was obtained within 1.5 h after spraying the virus aerosol solution onto the sensor electrode. Furthermore, our paper-based MIP sensor was easily integrated with a wearable face mask for the future detection of viruses in aerosols.

A few limitations regarding this MIP sensor still exist. It took 1.5 h to detect the lentivirus in aerosol form. In the future, the drying time of aerosols on sensing electrodes could be reduced by using a convection evaporation process, in addition to drying using diffusion [33,34] resulting in a 3–5-fold reduction in drying time. More importantly, by using a highly conductive PEDOT:PSS [poly(3,4-ethylenedioxythiophene)-poly(styrenesulfonate)] polymer material instead of polyaniline, we may be able to obtain an even better detection limit and faster response rate. This could also circumvent acidic pH problems with the virus molecular imprinting process. In our experience, high-conductivity grade commercial PEDOT:PSS showed ≥200 S/cm electrical conductivity compared to <1 S/cm obtained from polyaniline. PEDOT:PSS may result in a 200-fold improvement in sensitivity or response time of the virus MIP sensor. With these improvements, virus aerosol detection could be achieved in under 10 min, where the limiting factor is the drying time.

Further improvements could be made by integrating this sensor with artificial intelligence (AI) and machine learning. With a humidity sensor and the addition of an AI algorithm, the virus detection time could be significantly reduced. Lastly, the MIP and NIP sensing electrodes were connected to a multimeter via electrical wires, limiting their real-world use. This could be overcome by wirelessly connecting the wearable MIP and NIP sensing electrodes to a smartphone app or cloud server, employing the Internet of things (IoT) in healthcare.

## Data Availability

The data presented in this study are available on request from the corresponding author.

## Supplementary Materials

The following are available online at https://www.mdpi.com/article/10.3390/bios13090861/s1: Figure S1. The specific and non-specific binding of lentivirus on the sensor; Figure S2. The stability test of the PANI electrode on the PET paper substrate; Figure S3. Photo of the experimental setup for temperature and humidity experiments; Figure S4. Humidity and temperature effects on the normalized resistivity of the polyaniline sensing electrode; Figure S5. Aerosol spray characteristics; Figure S6. Sensor performance in the virus aerosol environment; Figure S7. SEM images of sensor surface after virus exposure; Figure S8. Photographic image of aerosol virus detection experimental setup; Figure S9. Resistance data over time for lentivirus detection from the liquid; Figure S10. Current–voltage relationship for the MIP electrode; Video S1. Spraying virus on polyaniline electrode; Video S2. Face mask sensor. Reference [35] is cited in Supplementary Materials.
