# Supplementary material for: Wearable Biosensor with Molecularly Imprinted Conductive Polymer Structure to Detect Lentivirus in Aerosol
Source: Biosensors (Basel). 2023 Aug 31;13(9):861. doi: 10.3390/bios13090861 (PMC10527467; doi:10.3390/bios13090861)
Supplement: Supplementary file 1 [file biosensors-13-00861-s001.zip › biosensors-13-00861-s001/biosensors-2513677-supplementary - for xml.pdf]

Wearable Biosensor with Molecularly Imprinted Conductive Polymer Structure to Detect Lentivirus in Aerosol

Jaskirat Singh Batra [1], Ting-Yen Chi [1], Mo-Fan Huang [2,3,†], Dandan Zhu [2,†], Zheyuan Chen [4], Dung-Fang Lee [2,3] and Jun Kameoka [4,5,*]

[1] Department of Materials Science and Engineering, Texas A&M University, College Station, TX 77840, USA
[2] Department of Integrative Biology and Pharmacology, McGovern Medical School, The University of Texas Health Science Center at Houston, Houston, TX 77030, USA
[3] The University of Texas MD Anderson Cancer Center UTHealth Graduate School of Biomedical Sciences, Houston, TX 77030, USA
[4] Department of Electrical and Computer Engineering, Texas A&M University, College Station, TX 77843, USA
[5] Graduate School of Information, Production and System Research, Waseda University, Fukuoka 808-0135, Japan
* Correspondence: jkameoka@waseda.jp
† These authors contributed equally to this work.

Supplementary Information

Supplement 1: Specific and Non-specific binding: Equations for trendline fit to sensor response

The specific binding and non-specific binding of lentivirus on virus MIP sensor were calculated from MIP and NIP values (Figure S1). Given the fact that the MIP data represents the total signal of the sensor, the NIP data represents the non-specific binding, and the (MIP - NIP) subtracted data represents the specific binding of the sensor, the trendline fits were obtained in equations S5—S8. In these equations, the resistivity ratio and the virus concentration (TU/mL) are denoted by the variables y and x respectively.

By using Equations 4 and S7, the limit of detection for lentivirus sensing by the MIP sensor was estimated to be 4181 TU/mL. As observed in Figure S1, the linear fit for non-specific binding was nearly horizontal with a slope of 0.047, and the y-intercept of 0.448 was close to the resistivity ratio of 0.534±0.013 on NIP and 0.557±0.006 measured on MIP at zero virus concentration. This confirmed that the non-specific signal (NIP) was almost constant, and it had a minimal contribution to the total sensor signal (MIP) at high lentivirus concentrations. The specific binding signal, represented by the difference between MIP and NIP, was caused by the specific binding of lentivirus on the sensing electrodes, which is depicted using quadratic fitting.

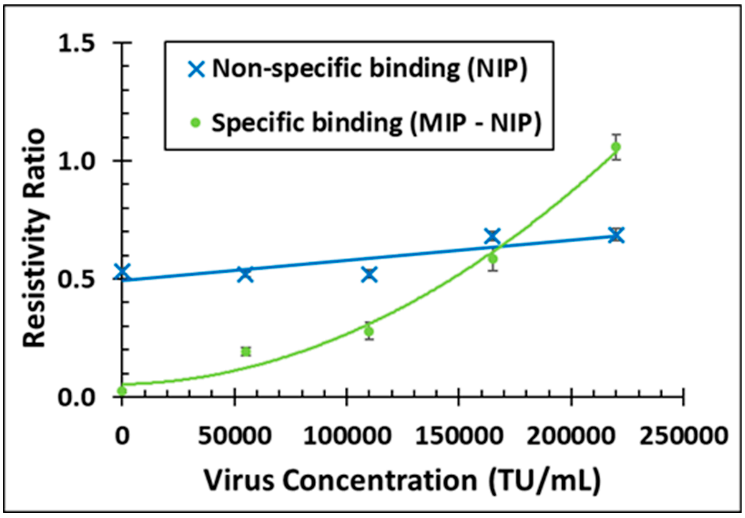

Figure S1. The specific and non-specific binding of lentivirus on the sensor. Lentivirus was detected after 30-min liquid saturation, aspiration, and 30-min natural drying process. Calibration curve for virus sensor is represented by the specific binding fitted trendline. (*n* = 3, RSD avg. = 7.7%) [RSD = Relative Standard Deviation]

Total sensor response is represented by:

$$y = 2.43 \times 10^{-11}x^2 - 1.26 \times 10^{-8}x + 5.77 \times 10^{-1} \quad \text{(S5)}$$

Non-specific binding is defined by:

$$y = 0.0471x + 0.4475 \quad \text{(S6)}$$

According to the specific binding subtracted data, the linear regression equation of the range from 0 to 2.2×10^5 TU/mL with the R^2 value of 0.91 is:

$$y = 4.47 \times 10^{-6}x - 6.44 \times 10^{-2} \quad \text{(S7)}$$

On the other hand, the quadratic regression equation for specific binding is:

$$y = 1.95 \times 10^{-11}x^2 + 1.78 \times 10^{-7}x + 5.38 \times 10^{-2} \quad \text{(S8)}$$

Supplement 2: Sensor Stability and Effect of Temperature/Humidity

The normalized resistivity of the polyaniline (PANI) electrode for 14 weeks is shown in Figure S2, where the resistivity of the PANI sensing element is normalized to Week 0. The normalized resistivity value remained stable (~1.214) for up to 11 weeks. From Week 12, the value increased by 25% which could be due to the change in polyaniline material characteristics. This test could evaluate the effectiveness of synthesis and the PANI material. The stability of PANI for 11 weeks may result from the hydrophobicity of the PET paper substrate that has better interaction with organic PANI and is more tolerant of

vigorous stirring during synthesis. The temperature and humidity changes did not have a significant impact on the sensor resistivity characteristics as discussed below. After 11 weeks, even though the normalized resistivity of the PANI electrode showed a slight increase, its effect on virus MIP sensor output may be removed by subtracting the MIP and NIP resistivity ratios. This is because both the electrodes of the sensor are made from the same PANI material.

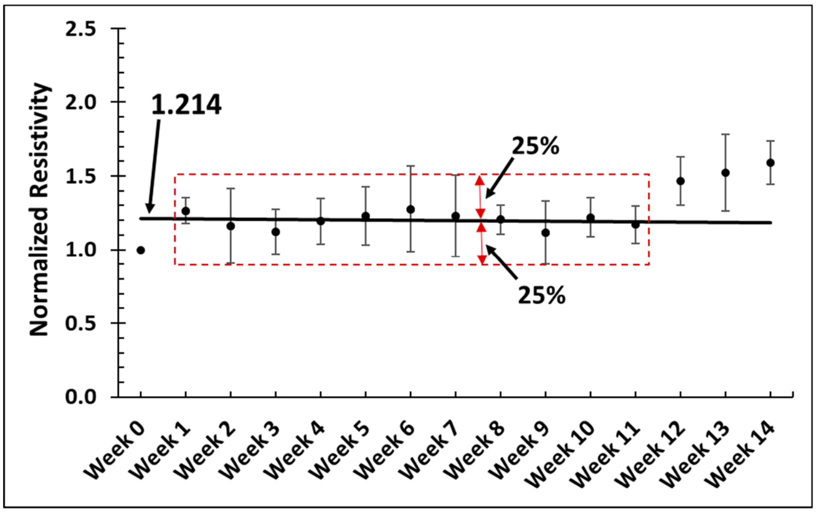

Figure S2. The stability test of the PANI electrode on the PET paper substrate. The standard deviations were calculated from the three measurements from the top, middle, and bottom sites on a single electrode. (n = 3, RSD avg. = 13.6%)

The influence of temperature and relative humidity on the electrical resistivity of the polyaniline sensing electrode was measured inside a closed chamber, and this chamber was connected to a humidity and temperature meter (THWD-5, Amprobe, WA, USA) to record the experimental conditions. Figures S3 (a) and (b) display the experimental setup with the chamber containing the sensor device and the sensing electrode connected to the multimeter on the outside. The temperature/humidity probe was connected to the chamber using an airtight seal. For varying relative humidity, the moisture-adsorbing desiccant (2-5mm Blue silica gel, Interteck Packaging, NY, USA) was used inside the chamber to decrease humidity (Figure S3a), or DI water was allowed to evaporate inside the chamber to increase humidity (Figure S3b). In the temperature experiment, cold packs were placed outside the chamber and covered with insulating polystyrene foam to decrease the chamber temperature. To increase the sensing electrode's temperature, the sensor device was removed from the chamber and directly placed on a hot plate.

(a)

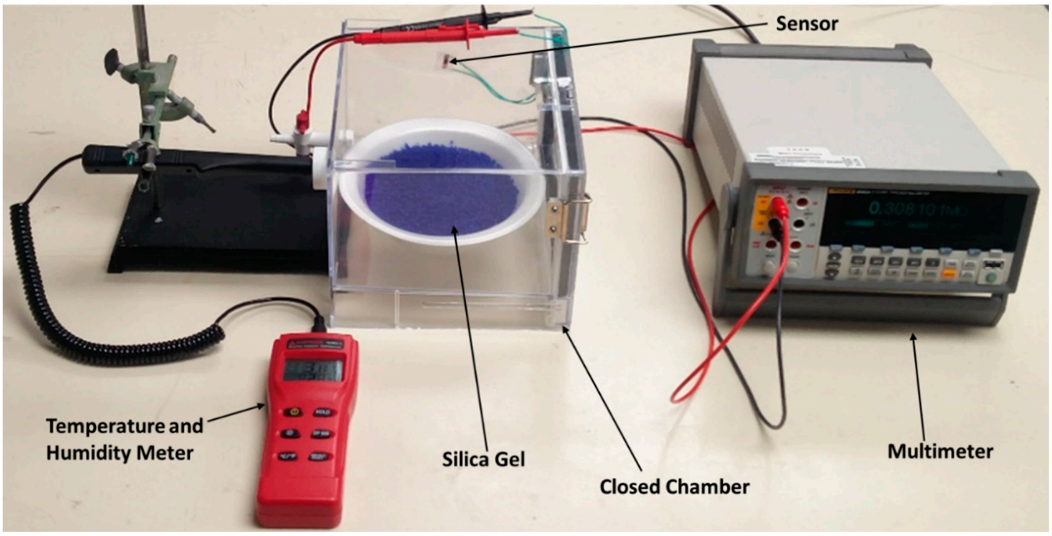

(b)

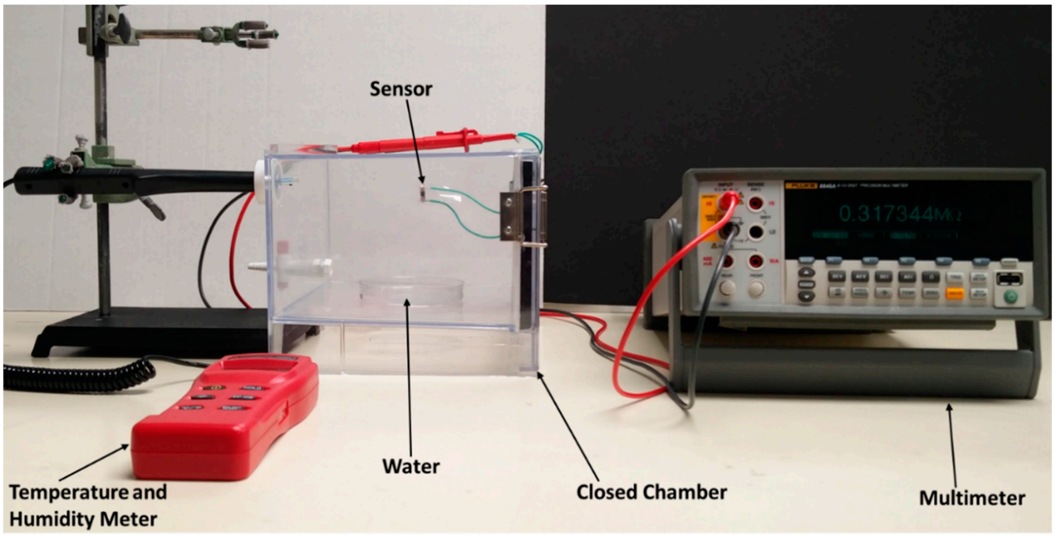

Figure S3. Photo of the experimental setup for temperature and humidity experiments. The temperature and humidity meter was connected to the chamber, and the multimeter was connected to the sensing electrode. (a) Silica gel was placed inside the chamber to decrease humidity, and (b) DI water was kept inside the chamber to increase humidity.

The electrical resistivity of the sensing electrodes measured at varying humidity and temperature condition was normalized to their values at room conditions (relative humidity 41.35 ± 0.78 %; temperature

22.7 ± 0.3 °C). The normalized electrical resistivity for varying humidity and temperature is presented in Figures S4 (a) and (b).

(a)

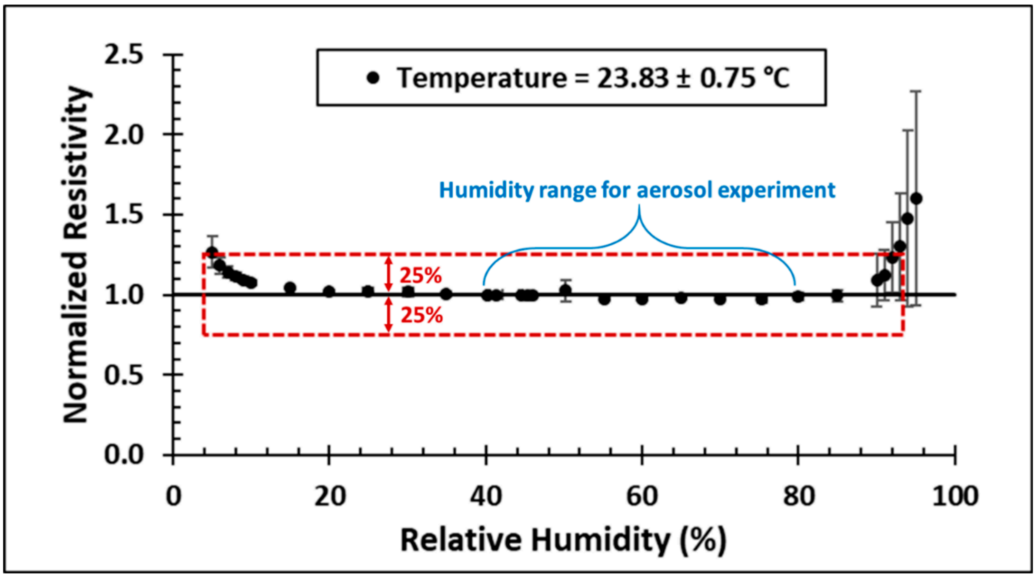

(b)

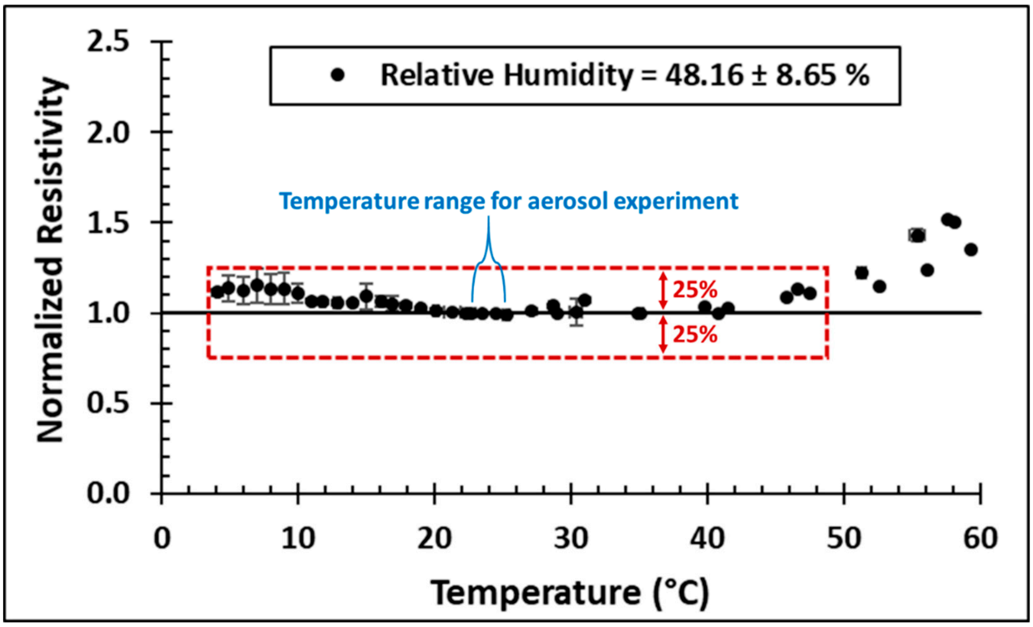

Figure S4. Humidity and Temperature effects on the normalized resistivity of the polyaniline sensing electrode, (a) Varying relative humidity, and (b) Varying temperature. (n = 3, RSD avg. = 4.8%)

In the humidity experiment, the normalized resistivity of sensor electrodes remained constant for relative humidity varying from 15% to 85%, and it showed a slight deviation outside this range. With a 25% deviation of normalized resistivity, the polyaniline sensor device could be used between 6% and 91% relative humidity conditions. Moreover, the normalized resistivity remained constant over the range of relative humidity conditions measured in most of our aerosol experiments, which confirmed that humidity did not play a significant role for the aerosol results shown in this paper. In some cases, we observed unstable resistivity ratios over time which is likely due to excessive moisture buildup in the air from continuous spraying inside the closed biosafety cabinet.

For the temperature experiment, the normalized resistivity was found to be approximately constant from 11.0 °C to 41.5 °C. The polyaniline sensor could be used between 4.1 °C and 47.5 °C temperature conditions with a 25% deviation in normalized resistivity. Further, the normalized resistivity was a fixed value over the temperature range that was measured for our virus aerosol detection experiments. These results proved that the temperature did not affect our virus detection.

Supplement 3: Aerosol spray characterization

To analyze aerosol distribution, a blue-colored dye was dissolved in DI water and sprayed onto a white sheet of paper from various spray distances (Figure S5a). The visual pattern formed by aerosols had an approximately circular shape, and the spray angle ϴ was calculated using the spray distance and the radius of the circular pattern. Spray angle as a function of distance is shown in Figure S5b. At the spray distances from 30 to 60 cm, the spray angle reduced sharply from 9.8±0.6° to 4.0±0.3°, while the radius of the circle showed a very little decrease from 5.21±0.25 cm to 4.25±0.20 cm.

The blue-colored aerosol sprayed on a white paper, which was marked with the sensor's fixed location, exhibited roughly circular patterns (Figure S5c). At a very short distance of 20 cm, which is outside the experimental range, the excess liquid was deposited that immediately flowed downwards. On the other hand, the spray at a 70 cm distance—also outside the experimental range—showed that the aerosol concentration was significantly decreased and very little volume reached this distance.

Within the experimental distance of 30 cm to 60 cm, the concentration of aerosol droplets decreased as the sprayer distance increased. This confirmed that lower aerosol volume reached distances far away from the sprayer. Even though liquid flowed downwards at the spray distance of 30 cm, similar to the 20 cm distance, this happened at a delayed time after spraying. Also, the volume that flowed downwards was smaller compared to the 20 cm case. Therefore, the 30 cm distance was included within the experimental

range. The aerosol concentration decreased significantly after a critical spray distance of 40 cm. Although no lentivirus was used for sprayer characterization, the spray patterns were crucial for theoretical calculations of lentivirus reaching the sensing element.

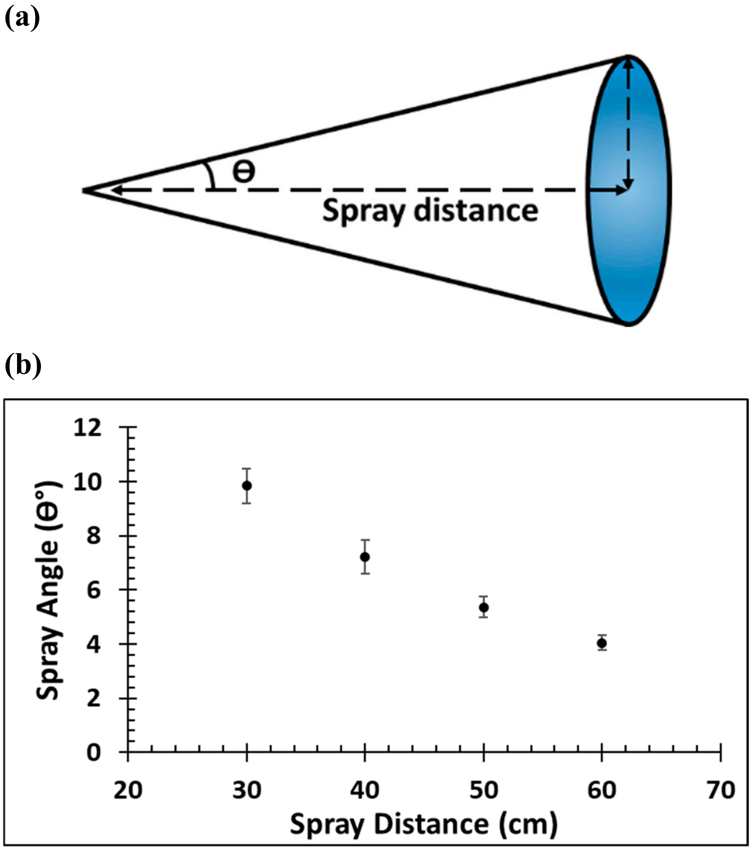

(c)

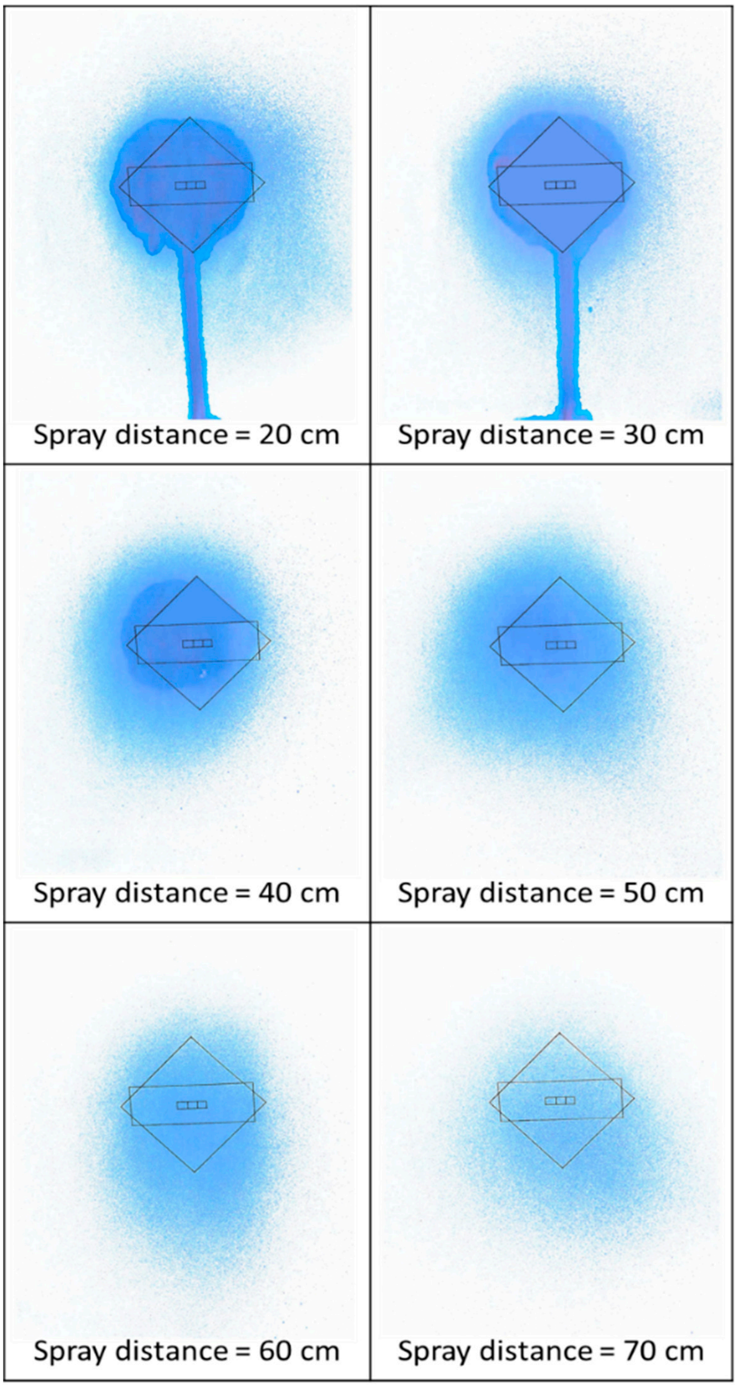

Figure S5. Aerosol spray characteristics (a) Diagram of aerosol spray distance and angle ϴ with the circular shape of the spray pattern. (b) Spray angle as a function of distance (n≥4, RSD avg. = 7.3%). (c) Visual image of patterns sprayed from 20 cm to 70 cm distances. Blue-colored dye was dissolved in DI water to visualize aerosols. Excess liquid at 20 cm distance and insufficient aerosols at 70 cm confirmed that the aerosols could only be generated within the 30 to 60 cm distance range.

Supplement 4: Sensor hybridization and detection efficiency in virus aerosol environment

Total number of lentivirus sprayed towards the sensor from various distances was ~5.8×10^5 TU. The virus amounts (and percentage of total) that reached the sensing element as well as detected by the sensor are shown in Figure S6a. As the spray distance increased, the sensor was moved farther away from the sprayer which resulted in a decreasing number of viruses reaching the sensor surface. Further, a much lower amount of the sprayed viruses were electrically detected by the sensor. This could be due to the electrical detection of only those virus particles that were trapped inside the molecularly imprinted cavities and not the ones that were non-specifically bound to the sensor surface.

The sensor's hybridization efficiency—a ratio of viruses detected to the viruses on sensing element, expressed as a percentage—was found to decrease with the increasing spray distance (Figure S6b). For spray distances between 30 cm and 60 cm, the virus hybridization efficiency was calculated to be 3.07% to 19.34%. The theoretical estimation for the amount of virus on sensing element is based on the assumption that all the virus aerosols sprayed from the source reached the sensing distance. However, the transport of virus aerosols in the air is far more complicated, and the actual number of viruses reaching the sensing element is less than the theoretical calculation. Thus, we can conclude that the sensor's hybridization efficiency is higher than the computed values in Figure S6b. The overall sensor detection efficiency—ratio of viruses detected to the total virus sprayed—was found to be between 0.33% and 2.90% for lentivirus sprayed from 30 cm to 60 cm distance.

(a) **(b)**

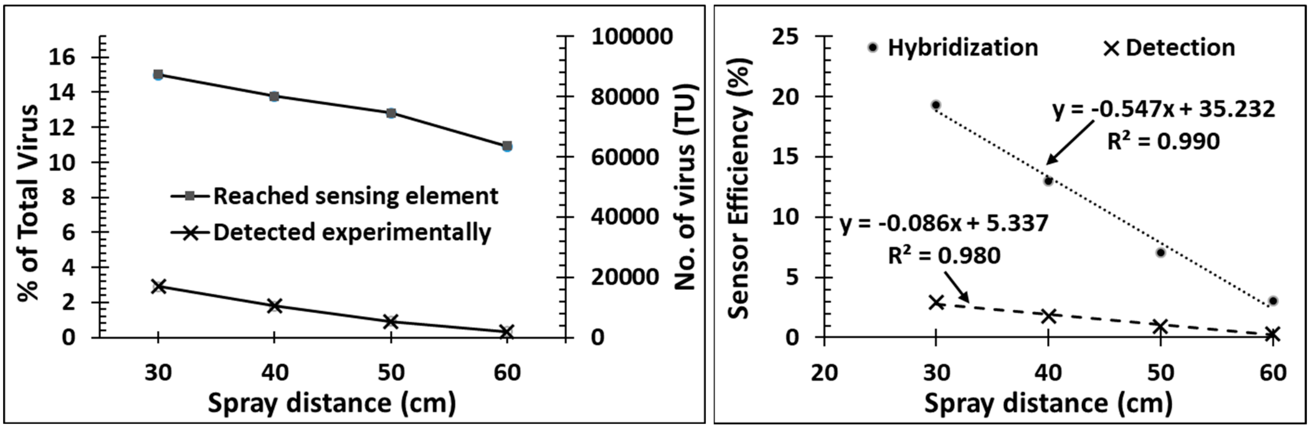

Figure S6. Sensor performance in the virus aerosol environment (a) Viruses reaching sensing element and experimentally detected as a function of sprayer distance. (b) Detection efficiency and minimum hybridization efficiency.

Supplement 5: Scanning Electron Microscopy (SEM) Images

The surface morphologies of MIP and NIP electrodes were investigated by a field-emission scanning electron microscope (LYRA3, TESCAN, Czech Republic). For the sample preparation, the MIP and NIP paper strips exposed to 2.2 × 10^5 TU/mL of lentivirus solution were firstly air-dried at 25 °C for 12 h, followed by vacuum drying for an additional 72 h. Subsequently, the samples were fixed on observation stubs by carbon tape and treated with Pt/Pd sputtering. The imaging was operated at 10 kV and a working distance of 9 mm.

The SEM images, having the same magnification, show the surface morphology of MIP and NIP sensing elements after virus detection in Figure S7a and Figure S7b, respectively. The surface of MIP (Figure S7a) presented several circular clusters of a diameter of around 200 nm. On the other hand, NIP (Figure S7b) structure showed larger irregular clusters textured with much smaller crystalline structures below 100 nm which were similar to the typical nanostructures of PANI as previously reported [35]. The smaller clusters on MIP could be attributed to the obstruction of cluster propagation caused by the lentivirus templates presented during the molecular imprinting process. For NIP, due to the absence of viruses during synthesis, the clusters can propagate to a larger domain without such an impediment. After virus detection, the lentivirus particles may have covered the molecularly imprinted structures as well as the nanocrystalline, resulting in the absence of natural textures of PANI in the MIP sensing element.

(a) **(b)**

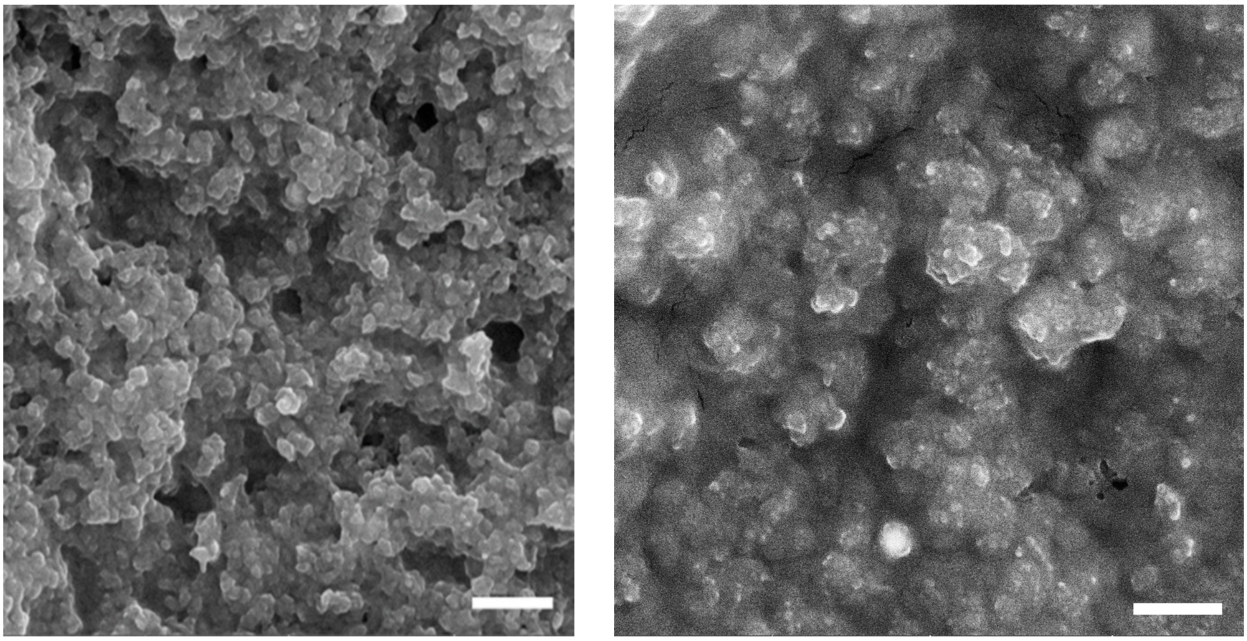

Figure S7. SEM images of sensor surface after virus exposure, (a) MIP surface, (b) NIP surface. The scale bar is 1 µm.

Supplement 6: Lentivirus Detection in Aerosol and Face Mask Sensor

The detection of virus in aerosols by spraying lentivirus aerosols on the PANI sensing electrodes was performed inside a closed biosafety cabinet (Figure S8). The aerosol volumes are controlled by the direct current (DC) voltage to the sprayer. To demonstrate the wearable virus sensor application, the lentivirus was also sprayed on a pair of NIP and MIP electrodes that were glued on a face mask via epoxy. The videos of aerosol spray and face mask virus sensing experiments are included in Supplemental Materials.

A battery-operated aerosol sprayer (Ultra-Low Volume Pistol Mist Sprayer, EcoVenger, NJ, USA) was converted to the DC power operation (1743B, B&K Precision, CA, USA) to control aerosol sprays from the outside of the biosafety cabinet. A voltage of 5 V can spray 2.65±0.15 mL of lentivirus solution (2.2 $\times 10^5$ TU/mL) at the rate of 0.25±0.01 mL/sec, and we sprayed for approximately 10-12 seconds for each experiment. Before spraying, the virus solution was kept in 15 mL centrifuge tubes which could be inserted directly into the detachable container of the aerosol sprayer. To detect the lentivirus in aerosol, the resistance measurements over time were recorded using a multimeter connected to the sensing electrodes. A metric ruler was used to calibrate the distance between the sensing electrode and the sprayer outlet.

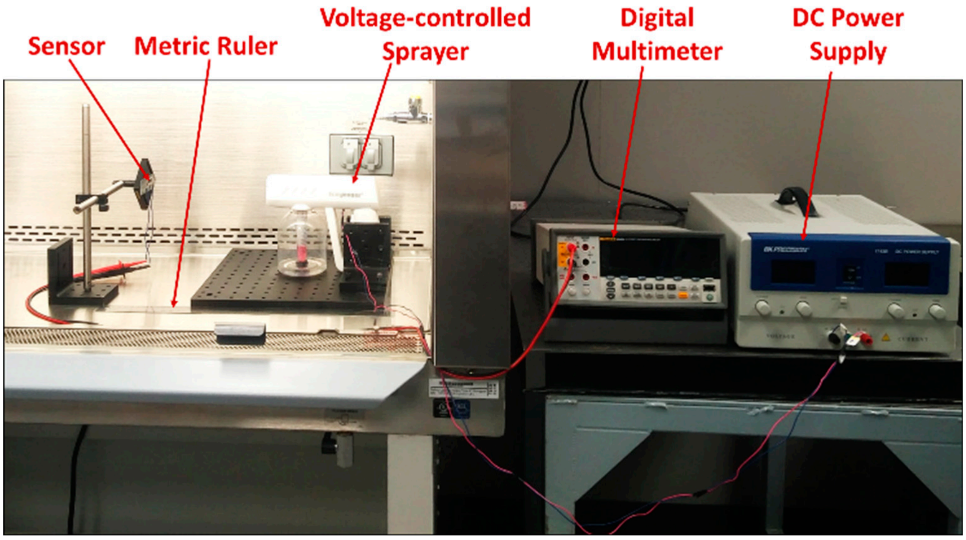

Figure S8. Photographic image of aerosol virus detection experimental setup. Virus aerosols were sprayed on the PANI sensor from distances of 30cm, 40cm, 50cm, and 60cm, and the resistance of the PANI sensing element was measured. *Video available for download from Supplements.*

Supplement 7: Resistance measurement over long time —lentivirus detection in liquid

The resistance data for lentivirus detection in liquid was measured directly using a multimeter. The time-response of MIP/NIP sensing electrodes for 2-hour and 24-hour duration is shown in Figures S9 (a) and (b) respectively.

Before the liquid droplet containing the virus was placed on the sensor, the dry electrodes showed stable resistance values. Once the liquid droplet (30 µl volume of ~1.65 × 10^5 TU/mL concentration lentivirus) was placed on a sensing electrode, at time zero, the resistance reading dropped rapidly. At 0.5 hours, the liquid droplet was removed from the electrode using a pipette and the resistance measurements were observed to slowly increase over time. The resistance readings reached the final saturation value at 1.5 hours and remained constant for 24 hours.

Even though the full saturation of resistance measurement was observed at 1.5 hours, the resistance value around 1 hour had reached at least 76% of the final measurement. Therefore, the 1-hour measurement time was used for faster virus detection in the liquid detection experiments.

(a) **(b)**

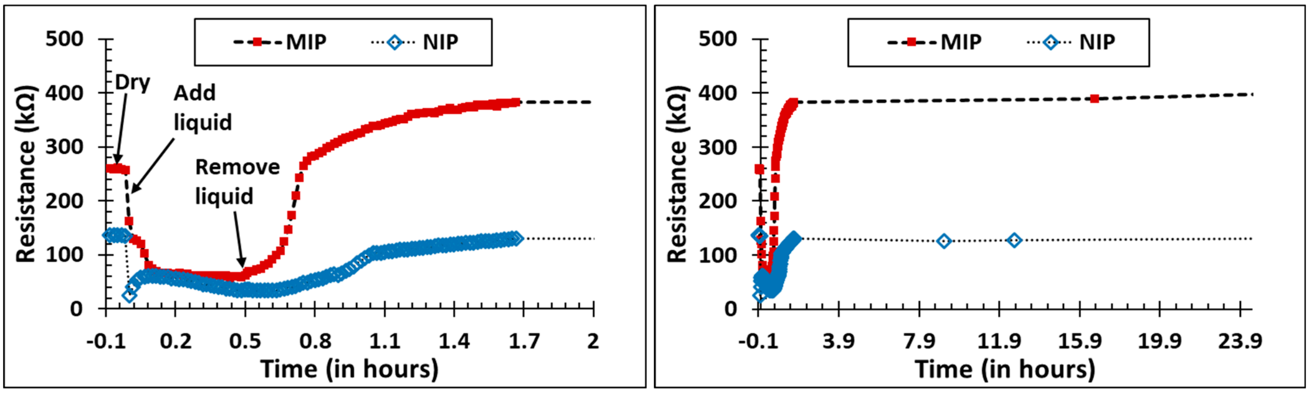

Figure S9. Resistance data over time for lentivirus (~1.65 × 10^5 TU/mL) detection from the liquid, (a) Measurements taken over 2 hours, (b) Measurements over the 24-hour duration.

Supplement 8: Current-Voltage electrical characterization

Using a sourcemeter (2450, Keithley, OH, USA), the current-voltage (I-V) relationship for the MIP electrode was obtained (Figure S10) for voltage range from -5V to +5V. As the voltage increased, the current also increased showing a positive relationship between V and I. Even though there is a slight deviation from linear fit around ±1 V to ±2 V, the MIP sensor behaved similarly to an electronic resistor with an approximately linear relationship between V and I. This confirmed that the resistance value of the MIP (and NIP) electrode could be used to detect lentivirus and calculate biosensor response.

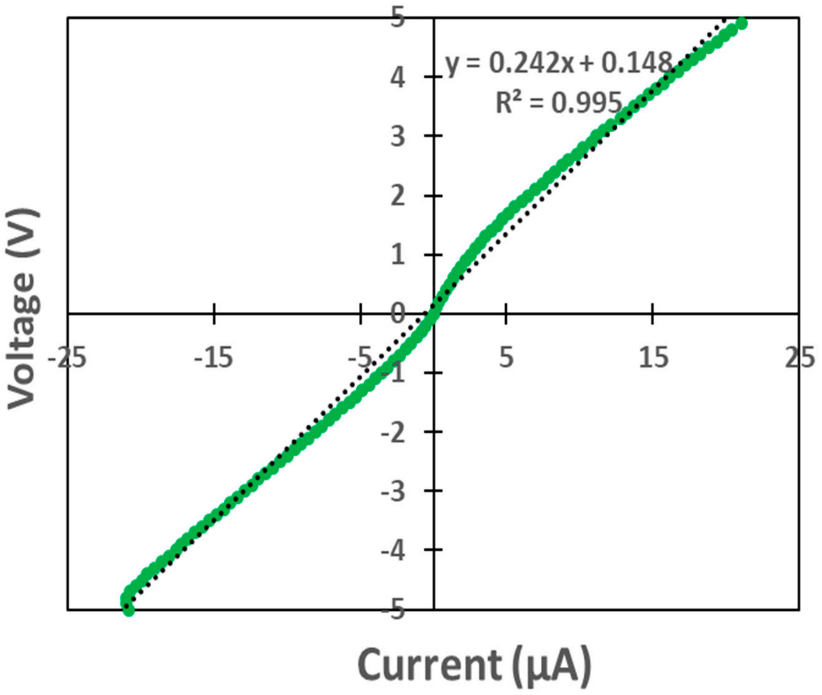

Figure S10. Current-Voltage relationship for the MIP electrode (-5V< V < +5V).

Before virus detection, the typical resistance values of polyaniline electrodes were between 1 kΩ to 300 kΩ, as evident from the slope of the V-I linear fit. In the future, polyaniline may be replaced with another highly conductive polymer, PEDOT:PSS [Poly(3,4-ethylenedioxythiophene)-poly(styrenesulfonate)], to further reduce electrical resistance and improve the sensitivity and detection limit of the molecularly imprinted sensor.

Supplement 9: Lentivirus Preparation

293T cells were seeded at 4 × 10^6 cells in Dulbecco's Modified Eagle's Medium (DMEM, Sigma, #D5671, St. Louis, MO, USA) complete medium containing 10% FBS (GenDEPOT, #F0900-050, Katy, TX, USA), 1% penicillin-streptomycin solution (100x) (GenDEPOT, #CA005-010, Katy, TX, USA), and 1% L-glutamine (GenDEPOT, #CA009-010, Katy, TX, USA) in a 10 cm-tissue culture dish. The medium was gently aspirated on the next day and added with 3 mL of opti-MEM medium (Gibco, #31985-070, Waltham, MA, USA). The plasmid mixture of 8 µg of pLKO.1 (Addgene, Plasmid #12273, Watertown, MA, USA), 2 µg of pMD2.G (Addgene, Plasmid #12259, Watertown, MA, USA), and 6 µg of psPAX2 (Addgene, Plasmid #12260, Watertown, MA, USA) was prepared in 300 µL of Opti-MEM medium. Then, 16 µL of Lipofectamine 2000 transfection reagent (Invitrogen, #11668030, Waltham, MA, USA) was taken to another Eppendorf tube with 300 µL Opti-MEM medium and incubated for 15 mins at room temperature, followed by adding the plasmid mixture. After incubation for 30 min, the plasmid-transfection reagent mixture was added to 293T cells and incubate for 3 h at 37 °C. Then, 10 mL of DMEM complete medium was added and refreshed after 12-16 h. The virus can be harvested at 48 and 72 h after transfection.
